# Metabolomic Characterization of Commercial, Old, and Red-Fleshed Apple Varieties

**DOI:** 10.3390/metabo11060378

**Published:** 2021-06-11

**Authors:** Adriana Teresa Ceci, Michele Bassi, Walter Guerra, Michael Oberhuber, Peter Robatscher, Fulvio Mattivi, Pietro Franceschi

**Affiliations:** 1 Laimburg Research Centre, Laimburg 6, Pfatten (Vadena), 39040 Auer, Italy; Adriana-Teresa.Ceci@laimburg.it (A.T.C.); michele_bassi@hotmail.it (M.B.); Walter.Guerra@laimburg.it (W.G.); Michael.Oberhuber@laimburg.it (M.O.); 2 Department of Cellular, Computational and Integrative Biology (CIBIO), University of Trento, Via Sommarive 9, Povo, 38123 Trento, Italy; fulvio.mattivi@unitn.it; 3 Research and Innovation Centre, Fondazione Edmund Mach, Via E. Mach 1, 38010 San Michele all’Adige, Italy; pietro.franceschi@fmach.it

**Keywords:** metabolomics, antioxidant activity, polyphenols, mass spectrometry

## Abstract

In this study, a metabolomic investigation was presented to correlate single polyphenolic compounds in apple pulp with quality characteristics such as antioxidant activity and content of phenolic compounds and anthocyanins in apple skin. Since the concentration of these compounds is influenced by environmental factors, the twenty-two apple cultivars originate from the same site. The polyphenolic compounds were analyzed by ultra-high-performance liquid chromatography coupled with triple quadrupole mass spectrometry (UHPLC-QqQ-MS/MS). The antioxidant activity, phenolic content, and anthocyanins were evaluated on the sunny and the shady sides of apple skin by spectrometric assays. In old apple varieties, the measured parameters were higher than in the commercial and red-fleshed varieties. By contrast, the profile of flavan-3-ols and anthocyanins was variable amongst commercial and red-fleshed varieties. The partial least square (PLS) method was applied to investigate the association between the skin proprieties and the metabolic profile of the pulp. The highest coefficients of determination in prediction (Q2) were obtained for compounds quantified in old cultivars. These results provided information to define the old apple varieties as a reliable group based on the pathway of the antioxidant compounds and anthocyanins content. Our results show the possibility to find cultivars with promising health features based on their content of polyphenols suitable for commercialization or breeding.

## 1. Introduction

Chronic diseases, cardiovascular disorders, cancer, lack of physical activity, and obesity are well known threats for public health [1]. The WHO (World Health Organization) reports that the number of deaths in the world linked to these issues is dramatically increasing due to environmental degradation, malnutrition, and behavioral risk factors [2].

Regular consumption of vegetables and fruits has been continuously linked to a health-promoting lifestyle [3,4]. The WHO encourages a minimum of 400 g of fruit and vegetables per day [1]. Indeed, fruits and vegetables are essential sources of a large class of valuable compounds such as polyphenols, vitamins, and minerals [5,6,7]. 

Apples (*Malus × Domestica*) are 12.5% of all consumed fruit in the world [8], and it is widely accepted that they are beneficial both to consumers in good health, and consumers with specific pathologies such as hypercholesterolemia [8], cancer [4], cardiovascular disease [9], pulmonary disorders, and Alzheimer’s disease [10]. Several studies highlighted the potential role of the antioxidants present in apples in preventing the formation of free radicals and reactive oxygen species (ROS) [11], which have been linked to chronic disease and inflammation. The most important antioxidants in apples are polyphenolic compounds such as hydroxybenzoic acids, esters of caffeic acid and quinic acid, and quercetin, especially in their glycosylated forms. The major class of apple polyphenols is the flavan-3-ols, accounting for 71 to 90% of the total polyphenolic compounds in apples, namely epicatechin, catechin, procyanidin B2, and a large amount of procyanidin oligomers, and subsequently, hydroxycinnamates (4–18%), flavonols (1–11%), and dihydrochalcones (2–6%). The anthocyanins represent 1–3% of the total phenolic compounds and contain cyanidin glycosides (3-galactoside, 3-glucoside, 3-arabinoside, 3-xyloside) [12]. The presence of high concentrations of these beneficial compounds can then be considered a key property in defining the “overall quality” of specific apple cultivars [13], and can be used to direct agronomic practices and specific breeding and selection programs. Another important class of polyphenols is that of the anthocyanins. They are hypoglycemic and decrease the risk of type II diabetes [14].

In apples, antioxidant compounds are distributed in the fruit pericarp (which is often consumed) and in the fruit peel. In both sections, their concentration is dependent on several agronomic, environmental, and genetic factors [15]. For this reason, agronomical and environmental conditions should be kept as uniform as possible to compare different apple cultivars in order to highlight potential genetic patterns [16]. To date, however, as reported by Simmonds et al. [17], there are a few studies that compare apple varieties cultivated in the same environmental conditions with identical agricultural practices. 

In this study, the different apple cultivars were categorized according to their phenotypic and commercial characteristics: old, commercial, and red-fleshed. Cultivars belonging to the old group originated between 1900 and 1950 according to Baric et al. [18], but they have lost popularity and consumer acceptance mainly due to deficits in productivity, homogeneity, storability, and/or shelf life [19]. The commercial group gathers the most consumed apple varieties currently available on the world apple market. Finally, the red-fleshed group includes a series of newly bred varieties characterized by a pigmented pericarp. This last group is considered particularly promising for future commercial exploitation for aesthetic and nutritional properties. The varieties present in this study are fingerprinted and validated based on the reference of Baric et al. [18] and they are commercially available [20]. 

Previous research by Farneti et al. [21] has suggested that the selection process of breeding and domestication did affect some, but not all, the phytochemicals contained in apples. However, also this extensive survey did not consider red-fleshed apples. While there is considerable interest in red-fleshed apples, due to the presence of the beneficial anthocyanins [22], it would be advisable to bring to the market new red-fleshed cultivar having both high concentration of polyphenols and anthocyanins. 

In this study, metabolomics was applied to characterize the polyphenolic profile of 22 apple cultivars belonging to the varietal collection of Laimburg Research Centre (Auer (Ora), Italy). All cultivars were true to type, accurately verified using molecular genetic tools [18]. To reduce the effect of the environment on the metabolic profile of the cultivars, all apple varieties were cultivated on M9 rootstocks in the same identical environmental using agronomic conditions. The cultivation area Trentino and South Tyrol were chosen for this study because they are market leaders in producing apples in Italy. Furthermore, they supply one tenth of apples in EU member states, with a lot of different varieties [23]. The present investigation focuses on the profiling of the antioxidant properties and of the polyphenol composition of the pulp and peel of apples. Furthermore, a multivariate partial least squares regression approach was used to investigate the cultivar-specific association between pulp and peel profiles. 

Our results showed that the variation in the level of phytochemicals is less pronounced in the old group, contrary to what was observed in the commercial apple varieties that demonstrate an extreme behavior. Furthermore, despite the fact that the red-fleshed apple cultivars belong to red-fleshed group, the concentration of metabolites is found to be extremely variable. 

## 2. Results and Discussion

### 2.1. Principal Component Analysis (PCA) Used as an Exploratory Tool to Evaluate the Overall Apple Sample Distribution

**Figure 1:**
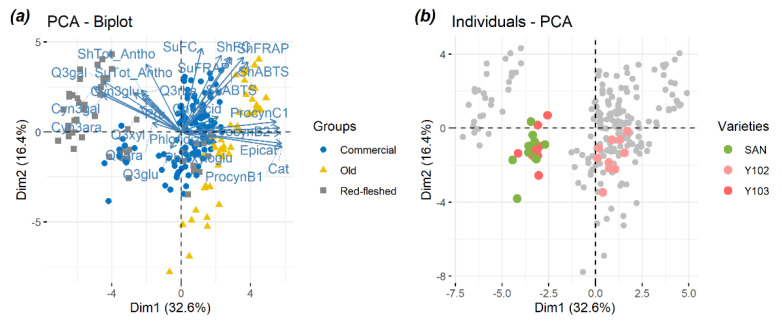
PCA of detected individual polyphenols in the pulp and of the antioxidant activities in the peel of 22 apple varieties. The colors (yellow, blue, and gray) indicate the apple classes (old, commercial, and red-fleshed). Loading is shown (**a**). PCA of the dataset where colors are used to highlight scores for three apple cultivars (one dot per apple shown): SAN, Y103, and Y102. The gray dots represent the other varieties. Scores are shown. (**b**). For apple variety names see Table 1.

To determine if the larger fraction of the variability in the concentration of phenolic compounds in apple pulp and the antioxidant activity in the peel could be associated to separation between the three apple groups (old, commercial, and red-fleshed), the full metabolomics dataset was analyzed using a principal component analysis (PCA). The results are reported in Figure 1. The PC1 vs. PC2 projection accounted for 49% of the total variance. In terms of the three apple classes (Figure 1a), the plot indicates that the three groups are separated enough. In particular, red-fleshed apples seem to be partially different from the others along PC1. The plot shows, however, the presence of one subcluster, and one red-fleshed is blended into commercial varieties. Indeed, the position of these three varieties in the PCA is highlighted in Figure 1b.

Looking at the old varieties (yellow color in Figure 1a), a general separation between cultivars was reported. Indeed, highly positive scores on the PC1 axis were observed, whereas the scores for other varieties were strongly negative on the PC2. In general, the loadings of these varieties showed a higher concentration of flavan-3-ols (catechin, epicatechin, procyanidin B1, procyanidin B2, and procyanidin C1) and antioxidant metabolites than the other samples. 

The commercial varieties (blue color in Figure 1b) occupied the center of graphs, indicating that the variability within this group was not responsible for a large fraction of the variance of the overall dataset. Despite of this actual trend, Santana (SAN) showed a notable separation from the others commercial cultivars blending with one red-fleshed cultivar. 

Regarding the old varieties, Wojdyło et al. [24] reported a good correlation between the antioxidant activity in vitro and the levels of procyanidins/flavan-3-ols. Thus, they supported our results, where a higher proportion of antioxidant and phenolic compounds was found in old cultivars compared to the other groups. Additionally, these results were consistent with other studies [12,25,26].

As in the case of old varieties, red-fleshed cultivars showed a high degree of separation. As far as the red-fleshed group (gray color in Figure 1a) is concerned, the plot indicates negative scores on the PC1 and highly positive scores on the PC2. Surprisingly, Figure 1b shows that the scores are negative both on the two PCs for Y103 and Y102 and they show an interesting separation from the other red-fleshed cultivars. The presence of different subtypes of red-flesh varieties has been proposed on the basis of phenotypic characteristics (type 1 and type 2; see Section 2.2). In terms of loadings, red-fleshed varieties had a higher concentration of anthocyanins (cyanidin glucosides) and flavanols (quercetin glycosides) than commercial and old cultivars, with Y103 and Y102 being a notable exception (Figure 1b). Indeed, Y103 and Y102 were categorized as red-fleshed type 2, but the concentration of quercetin glycosides, cyanidin glycosides, and flavan-3-ols was extremely variable. In contrast with the studies of Bars-Cortina et al. [27], where they identified that the Rni allele, controlling the red color, co-segregated with the MdMYB10 gene, our findings suggest that the red-fleshed metabolome was extremely variable. This variability led to a different distribution of polyphenolic compounds in apples, even though they are categorized as red-fleshed cultivars. The PCA for metabolites in apple pulp and peel highlighted an interesting negative correlation between flavan-3-ols and anthocyanins. This antagonistic behavior is in agreement with the literature, and can be explained with the biosynthetic pathway of phenolic compounds [28]. Indeed, the synthesis of flavan-3-ols and anthocyanins is regulated by anthocyanidin reductase and anthocyanins synthase, which are responsible for the inverse production of these two major phenolic classes. Furthermore, it is demonstrated that the concentration of anthocyanins is especially dependent on the sunlight exposure [29]. 

The general trends in the accumulation of metabolites in the cultivars were well understood [12], a closer investigation of flavan-3-ols and anthocyanins was undertaken. Thus, two in-depth investigations were conducted in this study, on the occurrence and distribution of flavan-3-ols and anthocyanins in apple pulps, and antioxidant activity in apple skin amongst these three variety groups [16].

### 2.2. Apple Pulp: The Polyphenolic Profile

**Figure 2:**
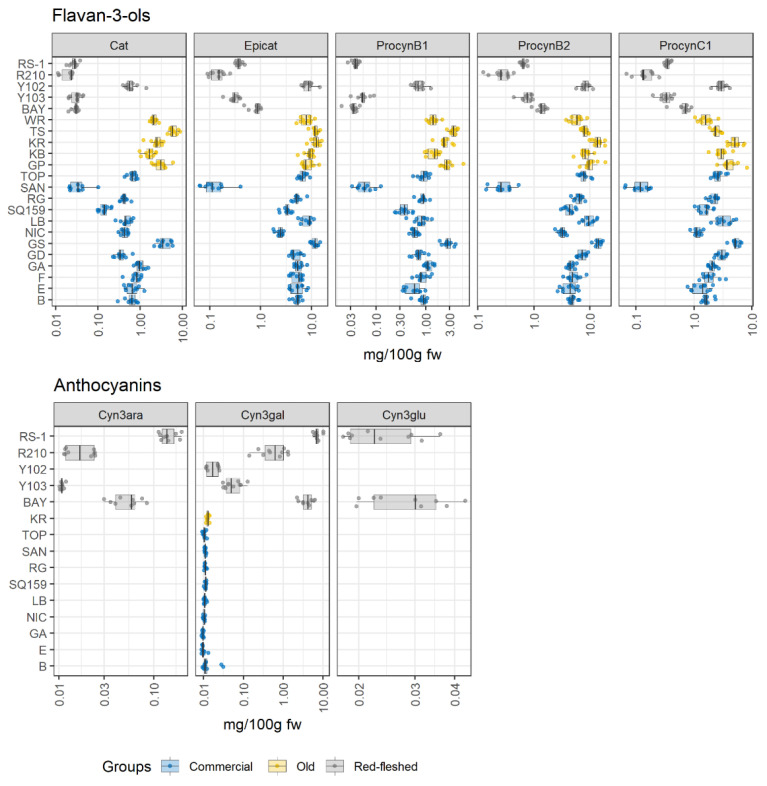
Concentration of flavan-3-ols and anthocyanins in the pulp of 22 apple varieties; 10 apples (biological replicates) per variety were analyzed. The concentrations of these metabolites were transformed in a log-10 scale. Varieties where anthocyanins were not detected have not been included in the graph. Cat = catechin, Epicat = epicatechin, ProcyanB1 = procyanidin B1, ProcyanB2 = procyanidin B2, ProcyanC1 = procyanidin C1, Cyn3ara = cyanidin-3-arabinoside, Cyn3gal = cyanidin-3-galactoside, Cyn3glu = cyanidin-3-glucoside, FW = fresh weight. For apple variety names see Table 1.

The flesh being the most widely consumed part of an apple, a detailed characterization of compounds in the pulp was performed. Figure 2 shows the concentration of flavan-3-ols (catechin, epicatechin, procyanidin B1, B2, and C1) and anthocyanidins (cyanidin-3-arabinoside, cyanidin-3-galactoside, and cyanidin-3-glucoside) in the different varieties organized in the three macrogroups. The results of both the quantification and of the statistical analysis are reported in Tables S1 and S3, respectively. 

The boxplot of flavan-3-ols clearly showed that the concentration of these metabolites was less variable in old apple varieties than in the other groups. In contrast, a marked variability in the concentration of flavan-3-ols amongst the commercial and the red-fleshed groups was observed, where several cultivars stood out. In both classes, the major flavan-3-ols could differ by up to two orders of magnitude within a group. In the case of the commercial group, SAN and Granny Smith (GS) showed the lowest and highest contents, respectively.

The other varieties seemed to show an almost uniform level of these phenolic compounds. As far as the red-fleshed group is concerned, Y102 significantly stood out among the red-fleshed apple varieties due to the highest concentration of all flavan-3-ols. In contrast, Y103 followed the same behavior as the other red-fleshed cultivars.

The boxplot of anthocyanins demonstrated that the red-fleshed apple cultivars contained the highest number of pigments as expected and previously mentioned in the literature [27,30]. The cyanidin-3-galactoside (cyn3gal) was also detected in a few apple varieties belonging to the commercial group and in one old apple variety; however, the concentration was low. Remarkably, cyanidin-3-glucoside (cyn3glu) and cyanidin-3-arabinoside (cyn3ara) were not detected in the red-fleshed Y102 variety. Bay 3484 (BAY) and RS-1, instead, contained the highest amount of all cyanidins quantified, and cyn3glu was only detectable in these varieties. The reason could be linked to the limit of quantification (LOQ) of the analytical methods employed for the analysis of these compounds. Remarkably, the concentration of anthocyanins varied among the red-fleshed apple varieties, even though all these cultivars are grouped as red-fleshed [31].

In summary, the work showed that catechin, epicatechin, and procyanidin B2 were the most abundant flavan-3-ols detected in all groups, followed by procyanidin B1 and procyanidin C1. In contrast, Belviso et al. [19] found lower amounts of catechin and epicatechin in their varieties under investigation, but our results are in agreement with those reported by Simmonds et al. [17]. As reported by Belviso et al. [19], cyn3glu was not detected in old and commercial varieties. Giomaro et al. [32] observed a smaller amount of cyn3gal in all of their red-fleshed apples and found that this metabolite was the most abundant anthocyanin in apples [30,31]. Differences in the concentration of metabolites should be expected, as they do not only depend on the genotype, but also on agronomic factors, the harvest time, and on environmental factors, which lead to considerable biological variation, even when the varieties are grown in the same orchard [19]. 

In relation to anthocyanins, the pigmentation of apples was correlated to the dominant red-flesh *MYB10* genes [27,30]. Chagné et al. [30] did not report differences in the protein sequence between red-fleshed varieties and GS, apart from its differential expression [30]. In general, the higher concentration of anthocyanins in red-fleshed varieties compared to white-fleshed varieties has been already reported by Bars–Cortina et al. [27]. On the other hand, the varieties Y103 and Y102 showed low concentrations of anthocyanins (cyn3glu was not detected), despite being red-fleshed varieties. A possible explanation for why only two varieties (Y103 and Y102) out of five matched the data reported by Bars–Cortina et al. [27] could be linked to red fruit-flesh phenotypes [30]. The type-1 red-fleshed apples were characterized by a red coloration in stems, roots, leaves, and whole fruits; instead, the type-2 red-fleshed apples showed the reddening only in apple peels and new leaves [27]. Thus, Y103 and Y102 could refer to the type-2 red-fleshed, and RS-1, BAY, and R210 to the type-1 red-fleshed based on the concentration of anthocyanins. 

Surprisingly, the red Y102 variety showed the lowest concentration of anthocyanins, but a high amount of flavan-3-ols. The occurrence and the distribution of flavan-3-ols and anthocyanins in the pulp of Y102 are similar like in the cortex of old apples varieties. This observation could be interpreted by considering the competitive pathway between flavan-3-ols and anthocyanins, which has been already reported by other studies [27,28]. These two phenolic subclasses utilized the same precursor: leucoanthocyanidins. Therefore, the competitive interaction of two different enzymes anthocyanidin reductase and anthocyanins synthase with the substrate could be responsible for differential biosynthesis of flavan-3-ols and anthocyanins, respectively [28].

It was noticed that the natural defense of apple trees against the apple scab infections (*Venturia inaequalis*) was linked to increment in the gene expression for the phenolic compounds [33]. Topaz (TOP) and Lb 17906 (LB), two scab-resistant apple cultivars, showed a considerable increment in the concentration of flavan-3-ols and these results were in agreement with the literature [33,34]. Noteworthy, Vanzo et al. [34] demonstrated that there is an upregulation in the polyphenolic pathway under organic production system both in scab-resistant and scab-susceptible apple cultivars. Therefore, based on the investigated substances, our results support the reasonable possibility to use the increment in the levels of flavan-3-ols could be potentially associated with the resistance of cultivars to scab.

Another important aspect is the potential allergenicity of the apples due to the presence of high quantities of Mal d 3 allergen [35]. Indeed, SAN, a scab-resistant and hypoallergenic variety, has the lowest concentration of flavan-3-ols and these results agree with Vanzo et al. [34]. Kootstra et al. [36] reported that the SAN cultivar caused lower allergic effects than TOP, also known as a hypoallergenic apple. Remarkably, differences in the concentration of flavan-3-ols in SAN and TOP were found in our studies. Indeed, Simonato et al. [35] studied the hypoallergenic activity of five old cultivars, and they found a great diversity in the levels of phenolic compounds. Thus, it is providing the consideration that the phenolic profile could hardly be used to define the potential allergenicity of an apple cultivar in apple allergic individuals [36].

Our findings suggest that the occurrence and distribution of polyphenols seems to be more homogenous in the pulp of old apple varieties. In contrast, commercial and red-fleshed cultivars demonstrate a noticeable variation in the concentration of phenolic compounds.

### 2.3. Apple Skin: The Antioxidant Activity (AA), Total Polyphenolic Content (TPC), and Total Anthocyanins Content (Tot Antho)

**Figure 3:**
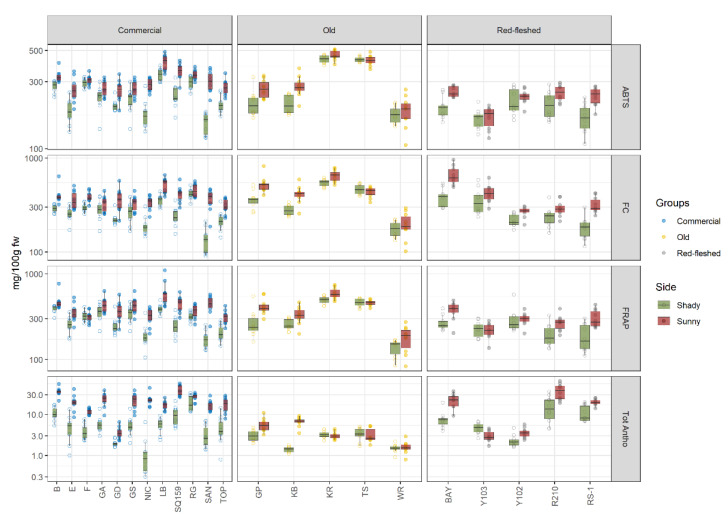
Results of FRAP, ABTS, FC, and Tot Antho in 22 apple varieties organized in three groups (old, commercial, and red-fleshed); 10 apples (biological replicates) per variety were sampled and the sunny and shadow exposed site of the apples were analyzed. The concentrations of these parameters were transformed in log-10 scale. FW = fresh weight. For apple variety names see Table 1.

The content of substances in apple skin is dependent on several factors, such as agronomic and environmental, and genetic ones [37]. This could imply that the response to sunlight exposure changes within cultivars. Figure 3 shows the results of 2,2’-azino-bis (3-ethylbenzothiazoline-6-sulfonic acid (ABTS) and ferric-reducing antioxidant power (FRAP), which are used to evaluate the AA, and furthermore Tot Antho and TPC (measured with Folin–Ciocalteu (FC)) in the 22 apple varieties organized in three groups: old, commercial, and red-fleshed. The results of both the quantification and of the statistical analysis method are reported in Tables S2 and S3, respectively.

Old, commercial, and red-fleshed apple varieties showed a comparable trend in the AA and TPC, as evaluated by FRAP, ABTS, and FC, contrasting with the observation in the pulp.

Regarding the old group, Tiroler Spitzlederer (TS) and Kanada Renette (KR) had the highest antioxidant activity and phenolic content. In contrast, Weisser Rosmarin (WR) showed the lowest content of antioxidants and phenolic compounds. Goldenparmäne (GP) and Kalterer Böhmer (KB) presented a marked difference in the concentration of antioxidants and phenols between the sunny and the shady sides. In contrast, the plot of commercial apple varieties provided clear evidence that there was a pronounced difference on the antioxidant activity and phenolic content between the sunny and shady sides of apples. Indeed, this difference was more noticeable in Golden Delicious (GD), Nicoter (NIC), LB, SQ159 (SQ159), SAN, and TOP. In detail, these two sides of the cultivars SAN and NIC were in marked contrast. In addition, the influence of sun has a less noticeable effect on the distribution of antioxidant and phenolic compounds in the apple skin of the varieties Braeburn (B), Elstar (E), Fuji (F), Gala (GA), GS, and Rosy Glow (RG).

The plot of red-fleshed cultivars illustrates that only the skin of BAY was influenced by the sunlight, showing a higher pronounced effect on antioxidant activity and the content of polyphenols [37].

The total anthocyanin content varied markedly between sunny and shady sides amongst all the three groups, where several cultivars stood out. Again, a more noticeable effect was observed in the commercial apple cultivars. The results of NIC stood out among the others, with a difference of almost two orders of magnitude in the total anthocyanin concentration. In contrast, the content of anthocyanins in the skins of old apple cultivars was less influenced by the sunlight, except for KB. Indeed, NIC and KB were bicolored varieties with a red overcolor on the sunny side [38,39]. Y103 and Y102 showed the lowest content of total anthocyanins and the exposure of fruits to the sun had a smaller effect on the skin pigmentation. In contrast, BAY, RS-1, and R210 have a higher content of anthocyanins, which varied considerably amongst these three cultivars. Since the peel is an important barrier protecting fruits from environment stress, these results suggest that exposure to sunlight leads to changes to the concentration of metabolites, even though the extent of the effect strongly depends on the variety. This observation is in agreement with previous studies [27,37] and can be rationalized with enzyme activities that are modulated by the exposure of fruits to the sun [27]. Furthermore, a higher level of anthocyanins in the peel than in the pulp is found, and this difference in the concentrations of phytochemicals in these different parts of apples is in accordance with other studies [25,40,41].

In general, the shady side of apples always showed a lower antioxidant, total polyphenolic, and total anthocyanin content. Indeed, the photosynthetic system and enzymatic activity is higher on the sun-exposed side than the shady side due to the upregulation of enzymes [37]. The overall upregulation can be interpreted considering the photoprotective role of flavonoids [37], which contribute to the antioxidant activity, total polyphenolic content, and total anthocyanin content [26]. Since the concentration of phenylalanine and tyrosine, which are the most important precursors in flavonoid biosynthetic pathways in apples, has been found higher in sun-exposed peels [37], this higher concentration of the pathway precursors can be linked to the observed results. Our findings, however, suggest that some varieties are able to preserve biochemical characteristics despite the exposure to sunlight, especially those belonging to old and red-fleshed groups. 

### 2.4. Prediction of the Antioxidant Activity in Apple Skin Based on the Polyphenolic Composition of Apple Pulp

Partial least square (PLS) regression was used to investigate the extent of the metabolic “association” between apple peel and apple pulp in three cultivar groups. The rationale behind this idea is to evaluate whether and to which extent the polyphenolic profile of the pulp can be used to predict the chemical characteristics of the skin. Separate models are then built and validated for the different parameters of the skin (ABTS, FC, FRAP, Tot Antho) in the three different variety groups for sunny and shady apples sides. 

**Figure 4:**
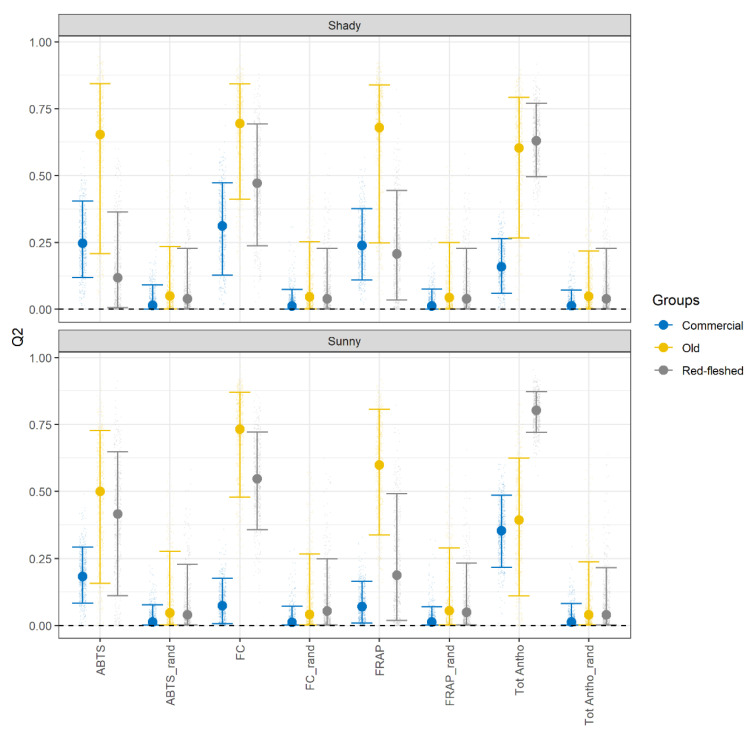
The quality of the PLS regression prediction model is measured by Q2 values using a 7-fold cross-validation (CV) repeated 10 times. The procedure of generating data, the splitting in train and test set, and fitting the models was set at 500 items. The points represent the median and the bars show the confidence intervals of 500 Q2 values. The confidence interval for the data is set at 99%.

Since the antioxidant activity depends on the assay used, ABTS and FRAP are separately treated [28]. The FC assay is used to evaluate the conformity between the TPC in peel and polyphenolic profile in pulp. The results are shown in Figure 4 in terms validation R2 (Q2) of the model.

In the plot, the Q2 of each model is compared with the predictive power of a model constructed on the same data in random order. When the confidence intervals of the data in the original dataset are overlapped to the confidence intervals of the data in the randomized dataset, the model was considered unable to perform a reliable prediction. 

It is important to mention that the variability of Q2 is expected to be dependent on the number of apples per group, so fewer variable results were expected (and observed) for commercial varieties (12 cultivars, 10 biological replicates, 120 apples). 

The plot of old varieties (yellow bars) clearly indicated that the PLS model showed a good predictive power for almost all assays considered, with median values of Q2 ranging from 0.5 and 0.7. A lower effectiveness was observed for ABTS and total anthocyanins content where the models did not perform better than random. For old varieties, a metabolic association between peel and pulp was supported. The situation was different for the commercial group (blue bars). In this case, PLS showed a lower effectiveness in predicting the properties of the peel. The confidence intervals for the predictions were narrower due to the higher number of samples, but Q2 never exceeded 0.5. In particular, the total anthocyanins content was only predicted reliably on the sunny side, whereas the results of other variables were predicted poorly. These results suggested a weak correlation between peel and pulp, which could be the sign of the presence of a more diverse metabolism within the commercial macro group. 

In the class of red-fleshed cultivars, Q2 values varied from 0.1 to 0.7, indicating a large variability in the prediction ability (gray bars). These findings allowed us to emphasize once again that despite that the red-fleshed cultivars being grouped into one class, the concentration of phytochemicals was strongly variable. Figure 4 highlights the marked variability in the prediction of ABTS, FC, FRAP, and Tot Antho in the red-fleshed group. In contrast, the variation in the prediction ability is less pronounced in the old and the commercial groups, even when the median values of Q2 are low in the commercial varieties. 

Looking in detail at the different parameters, the prediction of ABTS was achieved—even with different levels of accuracy—in the commercial and old groups, in contrast to what was observed in red-fleshed cultivars. PLS, instead, showed an efficient prediction ability for FC values in almost all groups, except for the sunny side of the commercial group. The prediction of FRAP was weak in the shady side of apples in commercial varieties and the sunny side of old varieties. Finally, the model was always able to predict the anthocyanin content, even when the median values of Q2 were low, such as in the commercial varieties. It is worth mentioning that the prediction of the total anthocyanin was more efficient in the sunny side than the shady side in the red-fleshed and the commercial groups, in contrast to what was observed in the old group. 

Lim et al. [42] tried to predict the antioxidant activity in Australian fruits based on a metabolomic approach. PLS was used by Sahin et al. [43] to determine the antioxidant activity in fruit juices using high-performance liquid chromatography. The effect of storage in changes of antioxidant activity and total anthocyanin content in fruits was studied by Zheng et al. [44] using the PLS method. In both cases, the studies reported a positive correlation between the total polyphenolic content and antioxidant activity, whereas the individual polyphenolic compounds were hardly considered [13,45]. In this study, we confirmed a positive correlation, but we highlighted that the shady or sunny sides and the genotype of the variety contribute to determine the model performance. 

Our results show that ABTS, FRAP, and FC assays followed different behaviors, even though they could be used to measure the AA and TPC in plant extract, respectively. Indeed, the ability of prediction was also highly variable between the sunny and the shady side of an apple. An exhaustive investigation was turned up by Csepregi et al. [46] regarding the main flavonoids that contribute to antioxidant activity in plant extract. They affirmed that the responses of a specific antioxidant assay depended on the functional groups bound on the main chemical structure of the polyphenols [46]. In addition, Tsao et al. [11] specified that the cis-configuration is more effective than the trans-configuration [27]. Furthermore, hydrogen bonds, which influenced the electron delocalization, could be responsible for a diverse response of an assay [13]. Tsao et al. [47] used the Pearson correlation to establish that flavan-3-ols were the most reliable contributor to the antioxidant activity in apples and that procyanidins B2 and epicatechin were the most promising antioxidant polyphenolic compounds both in the peel and in the pulp [27]. Furthermore, Vanzani et al. [45] reported that apple oligomeric proanthocyanins had a higher antioxidant activity than their monomeric forms (catechin and epicatechin) [45]. Another important result was reported by Rossetto et al. [48], where they affirmed a synergic effect given by several antioxidants. These studies showed that a change in the polyphenolic composition and chemical characteristic of a phenolic compound could lead to discord outcomes of different assays.

Our results were in reasonable agreement with these literature data, confirming flavan-3-ols as the main contributor to the antioxidant activity of apples. Our findings showed that the apple varieties belonging to the old group were the most reliable cultivars and a metabolic “association” between peel and pulp of five different old varieties was found. Indeed, the best prediction ability was achieved in the old group due to the highest level of antioxidants present, namely flavan-3-ols. 

Regarding the sunny/shady sides of an apple, Li et al. [37] reported that there is an upregulation of enzymes in the pathway of polyphenols due to their protective role against sunlight. In detail, the levels of anthocyanins, flavonols, and total phenolics were raised in the sun-exposed side of apples [37]. Another study showed that there was an increased concentration of carotenoids and quercetin glycosides in the sun-exposed peel [49]. Our results were not completely in agreement with these works; in fact, it is generally found that the Q2 values are higher in the sunny side than the shady side, but not in all groups. Indeed, our results suggest that the cultivar selection could influence the efficiency in the performance of the prediction model both in the sunny and the shady side. Given the abundance of anthocyanin pigments existing in red-fleshed apples, the prediction of Tot Antho was high in the sunny side in apples of the red-fleshed group [37]. Furthermore, the PLS regression model was able to predict Tot Antho on the sunny side of commercial apple varieties. In contrast, our method hardly predicted Tot Antho in old varieties, and it is reasonable to suppose that the pathway of flavan-3-ols was favored in these cultivars.

## 3. Materials and Methods 

### 3.1. Chemical and Reagents

Formic acid (LC-MS grade) was obtained from Merck KGaA (Darmstadt, Germany). Acetonitrile (LC-MS grade) and methanol (LC-MS grade) were purchased from VWR International Srl (Milan, Italy). Liquid nitrogen was purchased from Rivoira (Milan, Italy). Sodium fluoride, phosphoric acid, glacial acetic acid, TPTZ, ABTS, iron (III) chloride hexahydrate, Trolox, potassium chloride, (+)-catechin, sodium carbonate, F-C reagent, quercetin-3-xyloside, 3,4-dihydroxybenzoic acid, naringenin-7-glucoside, and phloridzin were purchased from Sigma–Aldrich (St. Louis, MO, USA). Quercetin-3-rutinoside and potassium peroxydisulfate were purchased from Roth (Karlsruhe, Germany). Quercetin-3-glucoside, neochlorogenic acid, cryptochlorogenic acid, cyanidin-3-galactoside chloride, and malvidin-3-glucoside chloride were purchased from Extrasynthese (Genay, France). 

(−)-Epicatechin, procyanidin B1, procyanidin B2, procyanidin C1, chlorogenic acid, cyanidin-3-glucoside chloride, cyanidin-3-arabinoside chloride, phloretin, quercetin-3-arabinoside, quercetin-3-galactoside, quercetin-4’-glucoside, and quercetin-3-rhamnoside were purchased from Phytolab (Vestenbergsgreuth, Germany). Sodium acetate anhydrous was purchased from CHEMSOLUTE^®^ (Renningen, Germany). Hydrochloric acid from Fisher Chemical (Pittsburgh, UK). Deionized water was from MilliQ apparatus (Millipore Corp., Bedford, MA, USA).

### 3.2. Fruits Samples

In total, 219 apples of 22 cultivars were analyzed in this study and all cultivars were true to type, accurately identified using molecular tools [18]. The apple trees were grown in the experimental orchard of Laimburg Research Centre in Auer (Ora, Italy) at 220 m a.s.l. on M9 rootstocks under identical climatic and agricultural conditions [23]. Ten fruits per cultivar were harvested from four different trees, considering their optimal harvest time between August and November 2015. Apples were collected from the central canopy of the trees, leaving the tops and the bottoms out. The fruits were stored at +2 °C for 10 days at normal atmosphere (95% RH) and at shelf-life conditions (at room temperature (RT) in the dark) for 3 days. The harvest days, cold storage, and shelf-life period are reported in Table 1. 

The apples were peeled and cut into three equal equatorial discs choosing the central one. From the sunny and shady sides of each apple, a piece of 3 × 3 cm^2^ were taken.

The central disc and both peel parts were immediately frozen separately in liquid nitrogen and freeze-dried using FreeZone Freeze Dry System (Labconco, MO, USA). All dried samples were milled to a fine powder using a commercial miller, packed, and stored in hermetic polyethylene bags at −80 °C up until the analysis.

### 3.3. Extraction of Samples

The extraction protocol used was adapted from Valls et al. [50]. Twenty-give mg of freeze-dried apple material were extracted with 1.83 mL of a mixture of water:methanol (80:20 *v*/*v*) containing sodium fluoride (100 mM) and acidified with H_3_PO_4_ (0.01 ng/µL). The mixture was shaken for 15 min and centrifugated at +5 °C at 14,000 rpm for 5 min. The supernatant was removed and stored at −80 °C until the analysis.

### 3.4. Analysis of Polyphenolic Profile of Apple Pulps

Each extract of apple pulp was analyzed one time by resulting in a complete dataset of 219 apple pulp analyses. A quality control sample (QC) was made by pooling of 100 µL of extract for each apple variety and the QC was injected every 10 analyses to control the absence of chromatographic drift. Stock solutions at a concentration of 2000 ng/µL for each phenolic standard were made in 50/50 Millipore water and methanol and then a mix solution at 50 ng/µL was prepared. This last one was serially diluted to working concentrations. The calibration curve of polyphenolic standards covered a range between 25 and 0.0025 ng/µL. The retention time, polarity, molecular weight, precursors, products, collision energy, RF lens, regression parameters, and linearity range of each compound was summarized in S4. As blank, 5 µL of Millipore water was injected every 15 analyses. The analyzed solution for each sample contained 5 µL of two internal standards, Malvidin-3-glucoside and Quercetin-4′-glucoside (0.1 ng/µL) to monitor the instrument performance, 45 µL of the extract (or working standard solution) and 50 µL of MilliQ water.

The UHPLC-QqQ-MS/MS analysis was performed using an UltiMate 3000 UHPLC system (Thermo Scientific, Waltham, MA, USA) coupled with a TSQ Quantiva (Thermo Scientific, Waltham, MA, USA) triple-stage quadrupole mass spectrometer. Separation of analytes was performed using a Hypersil GOLD™ HPLC (2.1 × 50 mm, 3 µm, Thermo Scientific, Waltham, MA, USA). The mobile phase was A (2.5% [*ν*/*ν*] formic acid in acetonitrile LC-MS analytical grade) and B (2.5% [*ν*/*ν*] formic acid in Millipore water). The gradient elution was 0–1 min (2.5% B), 1–10 min (16.5% B), 10–11.5 min (16.5% B), 11.5–12.5 min (23.5% B), 12.5–15 min (55% B), 15–15.5 min (95%), 15.5–17.5 min (95%), 17.5–18 min (2.5% B), and 18–21 min (2.5% B) with a consistent flow rate 0.4 mL/min. The column temperature and autosampler were set at +5 °C and +40 °C, respectively. The operations were controlled by the Chromeleon Chromatography Data System (CDS) (version 6.8) software and Thermo Xcalibur (version 3.0) software (both Thermo Scientific, Waltham, MA, USA). The source conditions were as follows: voltage 1500 V, vaporizer temperature 275 °C, capillary temperature 325 °C, sheath gas 40 arbitrary unit (AU), auxiliary gas 15 AU, sweep gas 2 AU, and collision gas (Argon) 1.5 mTorr. The operation was controlled by Thermo TSQ Quantiva (version 2.0) software (Thermo Scientific, Waltham, MA, USA). The acquisition of analytes was done in positive ionization mode with electrospray ionization (ESI) source. The operations were controlled, and the quantification was calculated using Thermo TraceFinder (version 3.2) software (Thermo Scientific, Waltham, MA, USA). The polyphenols were identified by retention times and selected reaction monitoring (SRM) of reference compounds. The method was able to detect 22 polyphenols compounds and a total number of 17 polyphenols was found and quantified in the apple varieties under investigation. The individual polyphenolic compounds were grouped into six phenolic classes [11,25]: flavan-3-ols (procyanidin B1, B2, C1, (+)-catechin, (−)-epicatechin), anthocyanins (cyandin-3-galactoside, cyandin-3-glucoside, cyanindin-3-arabinoside), phenylpropanoids (chlorogenic acid), dihydrochalcones (phloridzin, phloretin-2′-xyloglucoside), flavanones (prunin), and flavonols (quercetin-3-galactoside, quercetin-3-glucoside, quercetin-3-xyloside, quercetin-3-rhamnoside, and quercetin-3-arabinoside). The quantification was carried out by external calibration curves of each compound. The results were normalized for the weight and expressed as the milligram of each compound per 100 g of sample on fresh weight.

### 3.5. Spectrophotometric Assays of Apple Peels

#### 3.5.1. Total Polyphenolic Content (TPC)

The TPC was determined by the Folin–Ciocalteu method and adapted from Valls et al. [50]. Two hundred and fifty µL of deionized water and sixty µL of extracts was added to sixty µL of Folin–Ciocalteu reagent. The mixture was mixed at 12,000 rpm for 6 min at RT. Then, 630 µL of sodium carbonates (7.5% *w*/*v*) were added to the mixtures and they were mixed at RT for 90 min. The absorbance was recorded at 740 nm on a Cary 60 UV–Vis (Agilent Technologies, Palo Alto, CA, USA) spectrophotometer, and referred to a standard curve of catechin (range 0–150 ng/µL). The results were normalized for the weight and expressed as milligram of catechin equivalents per 100 g of sample on fresh weight.

#### 3.5.2. Total Anthocyanins Content (Tot Antho)

The Tot Antho was determined by pH differential method and adapted from Valls et al. [50]. Two dilutions of the same extract were made by adding 800 µL of potassium chloride (0.025 M, pH 1) to 200 µL of sample extracts and 800 µL of sodium acetate (0.4 M, pH 4.5) to 200 µL of sample extracts. The absorbance was recorded at 520 and 700 nm on a Cary 60 UV–Vis (Agilent Technologies, Palo Alto, USA) spectrophotometer. Tot Antho was calculated using Lambert–Beer law (ℇ = 26,900 L/mol/cm, MW = 449.2 g/mol) from the measures at pH 1. The results were normalized for the weight and expressed as milligrams of cyanidin-3-glucoside equivalents per 100 g of sample on fresh weight.

#### 3.5.3. Antioxidant Capacity Measurements (AA)

##### Antioxidant Activity, ABTS Assay

The AA was determined using Trolox equivalent antioxidant capacity (TEAC) assay and adapted from Valls et al. [50]. For the assay, 1970 µL of ABTS reagent was added to 30 µL of sample extract and the mixtures was incubated into dark for 10 min at RT. The decrease in absorbance was read at 734 nm using a Cary 60 UV–Vis (Agilent Technologies, Palo Alto, CA, USA) spectrophotometer, and referred to a standard curve of Trolox (range 15.6–250 µM). The results were normalized for the weight and expressed as milligrams of Trolox equivalents per 100 g of sample on fresh weight.

##### Antioxidant Activity, FRAP Assay

The AA was determined by FRAP assay and adapted from Valls et al. [50]. For the assay, 960 µL of FRAP reagent was added to 60 µL of sample extract and 180 µL of MilliQ. The mixtures were incubated into dark at +37 °C for 90 min. The decrease in absorbance was read at 595 nm using a Cary 60 UV–Vis (Agilent Technologies, Palo Alto, CA, USA) spectrophotometer, and referred to a standard curve of Trolox (range 15.6–250 µM). The results were normalized for the weight and expressed as milligrams of Trolox equivalents per 100 g of sample of fresh weight.

### 3.6. Statistical Analysis

#### 3.6.1. The Quantification Analysis

An exploratory data analysis was made to check normal distribution of data and to assess the equality of variance (Shapiro–Wilkinson test and Levene’s test). For all analyses carried out on polyphenolic metabolites in apple pulps and antioxidant activity in apple peels, the ANOVA assumptions were violated, even after mathematical log transformation of data due to the heterogeneous samples. The data were analyzed through multiple comparisons with Kruskal–Wallis nonparametric test to find differences between apple varieties. If the Kruskal–Wallis test was significant, a post-hoc analysis could be performed to determine which levels of the independent variable differ from each other level. A post-hoc test was using the criterium Fisher’s least significant difference. The adjustment methods included the Bonferroni correction. The significant differences were accepted by <0.05 and represented by different letters (S3). For this purpose, the following R package was used: agricolae [51].

#### 3.6.2. The Exploratory Tool: PCA

The dataset used for this purpose made of the data of chromatographic polyphenolic pattern of the apple pulp extracts—which presents multiple chemical compounds and their relative concentrations—and the data of AA, TPC, and Tot Antho of the apple peel extracts.

In the case of the LC/MS analysis, the dataset contained some missing values (S1), because certain compounds were not determined in apple pulp extracts caused by the concentration under the limits of quantification (LOQ). To prepare a complete dataset for statistical analysis, the missing values were handled by replacing them with random small values between zero and the limits of detection (LOD) [52]. 

In food chemistry studies, PCA is used to investigate where clusters within the samples exist in a dataset without assigning a prior membership [53]. To reduce the complexity of the dataset, the data were log10-transformed and scaled to unit variance to prevent some variables becoming dominant due to the greater quantity quantified. Then, the data were subjected to PCA [54]. 

In regard to PCA, based on the Kaiser criterion, the number of principal components (PC) is defined by eigenvalues higher than 1 which were considered “significant” in the data submission to the PCA analysis [53].

For this purpose, the following R packages were used: FactoMineR and factoextra [55,56].

#### 3.6.3. The PLS Regression Analysis

The PLS approach covers a huge number of applications in different scientific fields and this regression model is commonly used to predict specific properties from the chemical composition in matrices [57,58,59]. The PLS models used the X matrix (the pulp dataset) and Y matrix (the skin dataset) simultaneously, finding the latent variables in X that best predict the latent variables in Y. The X-matrix consisted of 17 × 219 elements (number of polyphenolic compounds in columns × number of samples in rows). The Y-matrix was modified for each assay. The Y-matrix was made of 1 × 219 (number of one AA assay in columns (FRAP, ABTS, FC or Tot Antho) × number of samples in rows. To define the metabolic correlation between peel and pulp, the complete dataset was used, and the data were log-10 transformed, mean-centered, and auto-scaled prior to the multivariate modelling.

The method validation was carried out by using a repeated 7-fold cross-validation (CV). Thus, the data were shuffled and random sampled multiple times to make a robust model that covered the maximum number of samples in training and testing operations. The K parameter was an integer value, and it indicated that the dataset would be split into K folds randomly. Among the K folds, the K-1 was the train dataset, and the model was trained on it. The train dataset was made of 70% of the complete dataset. The remaining dataset (20% of the complete dataset) was used as a test set, and it was used to evaluate the performance of the model. The CV was repeated 10 times to optimize the model parameters (such as the significant components) and the model was trained on the train set. The “goodness” of prediction of the optimized model was evaluated by predicting the Q2 value using the held-out test set. The statistical parameter named Q2 was used to demonstrate the ability of the PLS regression model in predicting the antioxidant activity, total polyphenolic content and the total anthocyanin content in apple peel based on the polyphenolic compounds quantified in apple pulp. In order to assess the variability of the results, the procedure of generating data, the splitting in train and the test and fitting models were repeated 500 times.

To validate the PLS models, one randomization sample was created by randomly assigning all measured values to each apple sample. The prediction was made as aforementioned and the Q2 generated from both dataset—the original dataset and the modified dataset—were compared. 

For this purpose, the following R package was used: caret [60]

## 4. Conclusions

Fruits and vegetables are rich in bioactive compounds, which are able to prevent many chronic human diseases; the consumption of apples has been continuously linked to lower risk of the onset of cancer [11]. Our results support the evidence that consuming different apples belonging to several cultivars could provide several classes of polyphenols beneficial to human health. This study drew attention to 5 old, 12 commercial, and 5 red-fleshed apple varieties. We demonstrated the utility of the metabolomic approach to determine that the old cultivars were a reliable group based on the metabolic pathways of the polyphenolic compounds, the antioxidant properties, and total anthocyanin content. Furthermore, we noticed that the variability in the concentration of polyphenolic compounds was more pronounced in commercial and red-fleshed apple varieties. In particular, for the red-fleshed varieties, it was interesting to observe that there were some genotypes (Y102, BAY and RS-1) combining a significant concentration of anthocyanins and a high content of the other classes of polyphenols in the pulp. Indeed, Y102 showed a higher amount of flavan-3-ols (21.74 mg/100 g FW) compared to the most consumed GD (16.42 mg/100 g FW). Additionally, 2.94 mg/100 g FW and 1.45 mg/100 g FW of flavan-3-ols in the BAY and RS-1 varieties were reported, respectively. Furthermore, anthocyanins content of 0.02 mg/100 g FW, 4.14 mg/100 g FW and 7.50 mg/100 g FW were found in Y102, BAY, and RS-1, respectively, whereas in the GD they were absent. Moreover, a noticeably content of total anthocyanins in the peel of the sunny exposed side of the apples were found, 3.65 mg/100 g FW, 20.01 mg/100 g FW and 23.03 mg/100 g FW in Y102, RS-1, and BAY, respectively. The data suggested the consumption of the whole fruit and these red-fleshed cultivars could be potentially used as new functional apples. These findings were in agreement with the results obtained by the prediction model. Indeed, we were able to demonstrate the reliability of the partial least square regression as a powerful method to predict the antioxidant activity in the apple skins. The efficiency at maximum precision was achieved in the old group confirming that the variability in the concentration of metabolites was less pronounced in the old group considered for this survey. 

During the last decades, consumers’ perception of the quality of fruit has been influenced by specific features, namely crunchiness, shelf life, and juiciness. Currently, these criteria have been used to steer the breeding process towards an improvement in the consumer acceptability, at the expense of healthy chemical compounds, such as polyphenols. Thus, it is potentially leading to the commercialization of new varieties with lower nutritional values. The phenolic profile of the apples has not been taken into consideration when applying the breeding process. Our results showed that the evaluation of the metabolomic profile of the apples is a suitable approach to assess the quality of fruits in the breeding programs or to evaluate new functional apples to the human diet. In conclusion, our findings suggest that the old varieties could be considered as potential fruits for apple breeding programs, encouraging the valorization and preservation of local cultivars, and thus confirming the results found by other studies [12,25,35]. The aim was to contribute new understandings towards selecting promising fruits with health features based on the metabolic pathways of the phenolic compounds and anthocyanins. 

## Figures and Tables

**Table 1:** List of cultivars, code of cultivars, variety group, harvest days, storage days, and sampling days of apples analyzed in this study (d = day, CS = cold storage, SL = shelf life).

Apple Cultivars	Code	Groups	Harvest Date	+10 d CS	10 d CS + 3 d SL
Goldparmäne	GP	Old	20 August 2015	30 August 2015	2 September 2015
Kalterer Böhmer	KB	Old	15 September 2015	25 September 2015	28 September 2015
Kanada Renette	KR	Old	15 September 2015	25 September 2015	28 September 2015
Tiroler	TS	Old	3 November 2015	13 November 2015	16 November 2015
Spitzlederer					
Weisser	WR	Old	28 September 2015	8 October 2015	11 October 2015
Rosmarin					
Braeburn	B	Commercial	2 October 2015	12 October 2015	15 October 2015
Fuji (Brak)	F	Commercial	6 October 2015	16 October 2015	19 October 2015
Elstar	E	Commercial	20 August 2015	30 August 2015	2 September 2015
Gala (Simmons)	GA	Commercial	12 August 2015	22 August 2015	25 August 2015
Golden	GD	Commercial	14 September 2015	24 September 2015	27 September 2015
Delicious					
Granny	GS	Commercial	24 September 2015	4 October 2015	7 October 2015
Smith					
Nicoter	NIC	Commercial	16 September 2015	26 September 2015	29 September 2015
Lb 17906	LB	Commercial	27 October 2015	6 November 2015	9 November 2015
SQ159	SQ159	Commercial	17 September 2015	27 September 2015	30 September 2015
Rosy Glow	RG	Commercial	27 October 2015	6 November 2015	9 November 2015
Santana	SAN	Commercial	12 August 2015	22 August 2015	25 August 2015
Topaz	TOP	Commercial	21 September 2015	1 October 2015	4 October 2015
Bay 3484	BAY	Red-fleshed (type 1)	13 August 2015	23 August 2015	26 August 2015
Red-fleshed 2/Y103	Y103	Red-fleshed (type 2)	13 August 2015	23 August 2015	26 August 2015
Red-fleshed 3/Y102	Y102	Red-fleshed (type 2)	1 September 2015	11 September 2015	14 September 2015
Red-fleshed 4/R201	R210	Red-fleshed (type 1)	30 September 2015	10 October 2015	13 October 2015
RS-1	RS-1	Red-fleshed (type 1)	20 August 2015	30 August 2015	2 September 2015

## Data Availability

The data presented in this study are available in article and supplementary.

## Supplementary Materials

The following are available online at https://www.mdpi.com/article/10.3390/metabo11060378/s1: Table S1: List of the apple cultivars (column A), polyphenolic classes (column D-I) and polyphenolic compounds (column J-Z) quantified in the pulp of the 22 cultivars included in the study (mean ± SD, n = 10). Results are expressed as mg/100 g FW, Table S2: List of the apple cultivars, the antioxidant activity (FRAP and ABTS), total polyphenolic content (FC) and total anthocyanin content annotated in the peel (sunny and shady sides of apples) of 22 cultivars included in the study (mean ± SD, n = 10). FRAP and ABTS are expressed as Trolox mg/100 g FW, FC are expressed as catechin mg/100 g FW, Tot Antho are expressed as cyanidin-3-glucoside mg/100 g FW, Table S3: Kruskal–Wallis nonparametric test, post-hoc analysis is using the criterium Fisher’s least significant difference, the adjustment methods include the Bonferroni correction results on the antioxidant activity, total polyphenolic content, total anthocyanin content, the polyphenolic compounds, and polyphenolic classes in the apple cultivars included in the study, Table S4: Compounds analyzed with UHPLC-QqQ-MS/MS in this study, the corresponding retention time, ESI polarity, molecular weight, precursor ions, product ions, collision energy, RF lens, regression parameters, and linearity range are reported.
